# NephroCheck data compared to serum creatinine in various clinical settings

**DOI:** 10.1186/s12882-015-0203-5

**Published:** 2015-12-09

**Authors:** Sahra Pajenda, Aysegül Ilhan-Mutlu, Matthias Preusser, Sebastian Roka, Wilfred Druml, Ludwig Wagner

**Affiliations:** Division of Nephrology and Dialysis, Department of Internal Medicine III, Vienna General Hospital, Waehringer Guertel 18-20, 1090 Vienna, Austria; Division of Oncology, Department of Internal Medicine I, Vienna General Hospital, Waehringer Guertel 18-20, 1090 Vienna, Austria; Division of Transplant Surgery, Department of Surgery, Vienna General Hospital, Waehringer Guertel 18-20, 1090 Vienna, Austria

**Keywords:** Acute kidney injury, IGFBP7, NephroCheck, TIMP-2

## Abstract

**Background:**

Acute kidney injury is frequently observed at the intensive care unit, after surgery, and after toxic drug administration. A rise in serum creatinine and a fall in urine output are consequences of much earlier injury to the most sensitive part of tubular cells located at the proximal tubule. The aim of the present study was to investigate the course of two cell-cycle arrest urinary biomarkers compared to serum creatinine in four clinical settings: ischemic reperfusion injury, cardiac failure, severe acute kidney injury, and chemotherapy-induced kidney injury.

**Methods:**

A recently developed bedside test known as NephroCheck measures two urinary parameters: insulin-like growth factor binding protein 7 (IGFBP7) and tissue inhibitor of metalloproteinase-2 (TIMP-2). The test is based on a sandwich immunoassay technique. The final test output, labeled AKIRisk, is shown as a numeric result.

**Results:**

This report revealed that [IGFBP7] · [TIMP-2] in urine rise rapidly prior to any change in serum creatinine. A unique feature of all four clinical settings is that a rapid decline predicts the recovery of kidney function. Besides, a subclinical kidney injury might be detected by the test.

**Conclusion:**

This bedside test detects biomarkers of renal injury. A rapid decline in AKIRisk was associated with the restoration of kidney function, whereas a prolonged high AKIRisk score was associated with end-stage renal disease. However, the dynamics seem to differ, depending on the cause and the extent of injury. Further studies will be needed to clarify the issue.

**Electronic supplementary material:**

The online version of this article (doi:10.1186/s12882-015-0203-5) contains supplementary material, which is available to authorized users.

## Background

Acute kidney injury (AKI) is common in patients at the intensive care unit and after surgery [1, 2]. Independent of the severity of injury, the patients are subject to a high risk of developing end-stage renal disease (ESRD) [3, 4]. Serum creatinine (sCr) is a late marker of AKI and is routinely determined for the assessment of kidney function [5]. The most challenging aspect of AKI is the absence of clinical symptoms because the trauma occurs in individual nephrons which are not monitored or registered by nociception. Clinical evidence is usually obtained more than 12 h later, based on changes in sCr and urine output [6, 7]. The diagnostic parameters reported thus far include neutrophil gelatinase-associated lipocalin (NGAL) [8], kidney injury molecule 1 (Kim-1) [9], cystatin C (Cys C), liver fatty acid binding protein (L-FABP), and interleukin 18 (IL 18) [10]. The usefulness of these markers in predicting AKI has been reviewed in detail [11, 12].

A novel test method, known as NephroCheck™, measures two small tubular cell-derived molecules, [IGFBP7] · [TIMP-2]. According to recently published data, this test possesses the highest sensitivity for detecting AKI at an early stage [13, 14]. Given the short test procedure of twenty minutes from urine collection to the outcome, it can be used at any clinical ward, outpatient clinic, or intensive care unit [13, 15].

IGFBP7 and TIMP-2 are markers of cell cycle arrest and possibly apoptosis, inflammation, and tubular cell repair [13, 16]. These cell biological conditions appear to be most relevant in the development of tubular cell injury, when the loss of cell polarity, brush border derangement, and cell sloughing might occur [17, 18]. Such injury may induce cellular repair mechanisms, but may also deflect the organism from normal repair towards maladaptive repair by reducing perfusion, causing inflammation and subsequent fibrosis. This may then lead to chronic kidney disease (CKD), which further predisposes the individual to recurrent acute kidney injury [17–19]. Such pathological conditions have been detected in sepsis-induced lesions in humans [20], and ischemic reperfusion injury (IRI) studies in animal models [21]. The pathology caused by antibiotics, contrast media or specific chemotherapeutic agents is mainly observed in tubular cells [6]. Depending on the severity of injury, both apoptosis and necrosis of tubular cells have been reported as part of the cisplatin toxicity profile, which includes direct DNA damage, inflammation, and the activation of specific pathways [22]. This is a limitation of cisplatin, which is known to be a highly effective chemotherapeutic agent for the treatment of cancer [23].

We evaluated the applicability of the test method in selected patients at our nephrology inpatient ward who were deemed susceptible to AKI, including those with preexisting CKD. The following entities were included: kidney injury following IRI during kidney transplantation (TX), AKI due to cardiac failure, severe AKI, and chemotherapy-induced kidney injury related to cisplatin. The course of [IGFBP7] · [TIMP-2] were monitored in comparison to serum creatinine.

## Methods

Sixty-nine consecutive patients were tested at the nephrology inpatient ward (*n* = 52) and the oncology ward (*n* = 17) of the University Hospital of Vienna from 2013 to 2014. Inclusion criteria were the following: age over 18 years, clinical signs of comorbidities constituting a high risk of AKI or evident AKI. Only patients admitted to the intensive care unit and the nephrology inpatient clinic were selected for testing. These included kidney transplant recipients for the evaluation of ischemic reperfusion injury. Preexistent chronic kidney disease was no exclusion criterion in the nephrology group.

Inclusion criteria in the cisplatin group were age higher than 18 years, no history of CKD, and the initiation of chemotherapy based on cisplatin. Written and oral informed consent to the study and publication of the results was obtained from all patients. The investigation was approved by the ethics committee of the Medical University of Vienna (approval number 1598/2013). The patients’ clinical data, demographics, medical history, medication and laboratory data were obtained from their medical files and the databases of the hospital. Representative patients (oncology inpatients, inpatients with more than four data points, and hospitalization for more than 4 days) were selected. Twelve patients were chosen for the report; a further four cases have been described in the supplement (Additional file 1: Table A).

NephroCheck measurements were performed every 24- to 48-h and were discontinued when either sCr reached baseline values or the NephroCheck score fell below 0.3. Endpoints of measurement were patient discharge, an anuric status, and outcomes such as renal replacement therapy (RRT) or death.

Analyses of urine samples for the biomarker combination of [IGFBP7] · [TIMP-2] were performed with NephroCheck™, a tool designed by a California-based biotechnology company (Astute Medical, San Diego, CA, USA). The Astute140TM meter is a device based on a fluorescence labeling technique, which detects fluorescent signals from the immunoassay and calculates concentrations of IGFBP7 and TIMP-2 from the inserted cartridge. The device converts the measured signals into a single number, defining the relative risk of the patient developing AKI. The final result, known as the AKIRisk score, is obtained within 20 min. Quality controls were performed as recommended and provided by the manufacturer.

Urine samples were tested immediately for all patients at the nephrology department. For those at the oncology department, a maximum delay of 2 h occurred between collection and testing. In case immediate analysis was not possible, the samples were frozen at −20 °C after centrifugation and stored. Frozen samples were thawed and warmed to room temperature, and the NephroCheck™ test was performed immediately.

## Results

**Fig. 1:**
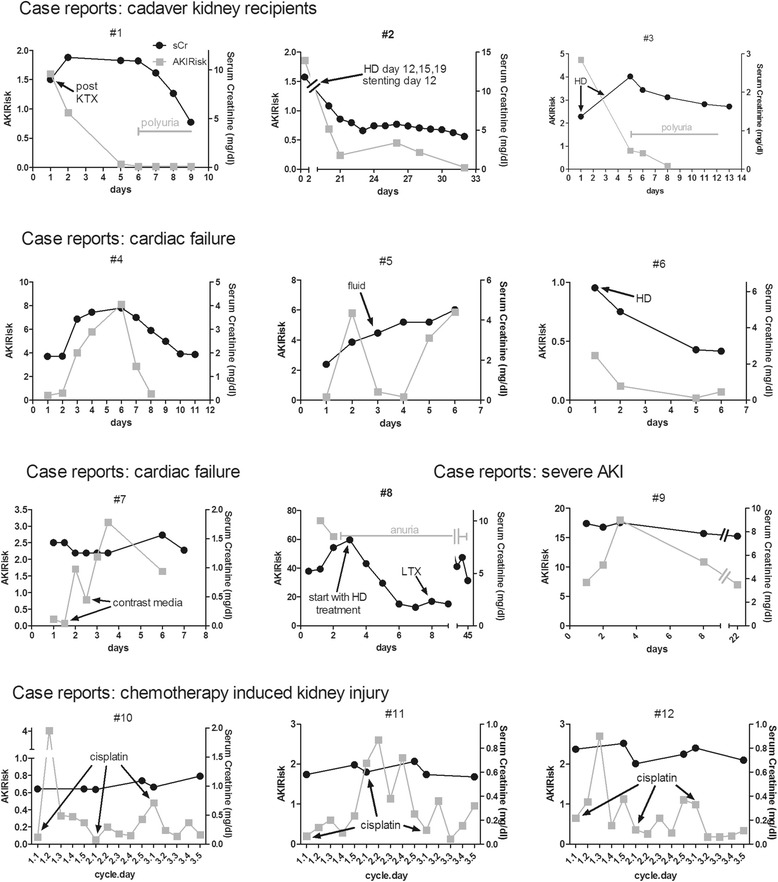
Dynamics of AKIRisk and serum creatinine. Time course (X- axis) of [IGFBP7] · [TIMP-2] labelled as AKIRisk on the left Y-axis (gray rectangles) and serum creatinine on the right Y-axis (black circles) in 12 patients within 4 different clinical settings. Case reports: kidney transplant recipients #1-#3; cardiac failure #4-#7; severe AKI #8-#9; chemotherapy induced kidney injury #10-#12. On the X-axis for the chemotherapy cohort the first digit represents the number of cycle, while the second digit is indicating the day of chemotherapy. sCr, serum creatinine in mg/dl; KTX, kidney transplantation; HD, hemodialysis; LTX, liver transplantation

Owing to the wide range of clinical settings, the patients’ data are presented in four groups consisting of individual case reports and graphic demonstrations in numeric order (#1- #12) depicted in Fig. 1. Demographic data and patient characteristics, including relevant information about the stage of AKI, peak AKIRisk and outcome, are shown in Table 1. Further four patients are shown in the supplements (Additional file 1: Table A, Additional file 2, Additional file 3: Figure A). Demographic data of the entire cohort, including AKI stages and the highest AKIRisk during the observation period are shown in the supplements (Additional file 4: Table B).

**Table 1:** Characteristics of patients

ID	Age	Sex	BSL sCr	BSL GFR	AKI Stage	Reason AKI	Peak AKIRisk	Outcome/sCr	Disease	Surgeries
1	66	m	11.29	4.5	End Stage	IRI	1.6	transplantectomy dialysis (0,5 m)	CKD, HTN	cadaver KTX
2	38	m	7.26	16.11	End Stage	IRI	1.86	3,48 (14 m)	CKD, HTN, mitral and aortic valve regurgitation	cadaver KTX
3	66	m	1.4	56.55	stage 3	prerenal, hypotension, shock	4.74	1,53 (19 m)	CKD, KTX, AKI, cardiomyopathy, afib	thoracotomy
4	69	f	1.21	44.12	stage 3	prerenal	8.11	1,37 (19 m)	CKD, aortic stenosis, CHD, HTN, NIDDM	Valve replacement, CPR, acute CABG
5	77	m	1.66	43.14	stage 3	prerenal, shock	5.86	Death	CTEPH, hyponatremia	none
6	70	f	1.77	31.58	stage 3	contrast media	0.38	1,58 (12 m)	CKD, CHD, HTN, afib, IDDM, diabetic nephropathy	none
7	73	m	1.3	64.63	No AKI	contrast media	3.12	1,26 (3 m)	HTN, mitral and aortic valve regurgitation, afib	none
8	56	f	unknown	unknown	stage 3	acute hepatits B	73	Lost to follow up	AKI, acute hepatitis B	LTX
9	78	m	8.2	6.79	End Stage	tubular injury membranous GN	18	5,48 (12 m)	CKD, HTX, HTN, NIDDM	none
10	41	m	0.93	98.18	No AKI	toxic insult	4.08	1,16 (2 m)	signet ring cell carcinoma of gaster	none
11	54	m	0.67	123.59	No AKI	toxic insult	2.61	0,52 (9 m)	Squamous cell carcinoma of the esophagus, HTN	none
12	57	m	0.73	107.46	No AKI	toxic insult	2.7	1,04 (13 m)	hypopharyngeal carcinoma	none

Demographics of the 12 patients depicted in Fig. 1 including age, sex, baseline GFR, stage of AKI, AKIRisk peak level during observation time, comorbidities and relevant surgeries before or during the observation period. The outcome such as follow-up creatinine levels are provided, with the months of follow-up presented in brackets. *BSL sCr* baseline serum creatinine in mg/dl, *GFR* glomerular filtration rate in ml/min, *AKI* acute kidney injury, *sCr* serum creatinine, *IRI* ischemia reperfusion injury, *GN* glomerulonephritis, *CKD* chronic kidney disease, *HTN* hypertension, *KTX* kidney transplantation, *CHD* coronary heart disease, *NIDDM* non-insulin dependent diabetes mellitus, *CTEPH* chronic thromboembolic pulmonary hypertension, *afib* atrial fibrillation, *IDDM* insulin dependent diabetes mellitus, *HTX* heart transplantation, *CPR* cardiopulmonary resuscitation, *CABG* coronary artery bypass graft surgery, *LTX* liver transplantation

### Cadaver kidney recipients

Patient #1: A 66-year-old man received a cadaver kidney organ with a cold ischemia time of 12 h. After transplantation the patient was observed briefly at the intermediate intensive care unit and then transferred to the internal ward, where urine output monitoring was started. The patient had been anuric for the preceding 6 months, requiring constant hemodialysis (HD). Oliguria was observed from day 1 to 4, followed by a phase of polyuria starting on day 5 with a decrease in sCr. Interestingly, the AKIRisk score on the NephroCheck™ test was high (1.6) when the patient’s initial urine output started, and fell rapidly to levels below 0.06 prior to the phase of polyuria. Two weeks after transplantation the patient needed a transplant nephrectomy due to rupture of the renal artery.

Patient #2: A 38-year-old man, oliguric and on dialysis for the last 2 years, received a cadaver kidney organ from a 15-year-old female non-heart-beating donor. After transplantation the patient was observed briefly at the surgery ward and then transferred to the internal ward (day 1) for further monitoring. The patient was oliguric to anuric during day 1–16. Donor organ perfusion and urinary tract morphology were monitored on a daily basis. Hemodialysis had to be performed three times from day 12 to day 19 post-transplantation. The patient needed stenting of the ureteral ostium to the bladder on day 12, and percutaneous drainage of the transplanted kidney pelvis. A percutaneous kidney biopsy revealed tubular cell damage, but no signs of graft rejection. TIMP-2 staining of tubular cells obtained from the urinary sediment on day 25 is shown in the supplement (Additional file 5: Figure B). Finally the patient was discharged 40 days post-transplantation, after a long period of gradually increasing urine volume. The AKIRisk score was high on day 1 (1.86), but could not be evaluated until day 20 because of bladder injury and constant bladder flushing with normal saline. The patient’s AKIRisk parameters were measured from day 20 until discharge, which decreased from 0.69 to 0.03. Urine output increased, accompanied by a fall in sCr.

Patient #3: A 66-year-old male kidney transplant patient experienced a hemorrhagic shock and lost his transplant function, requiring dialysis on day 1 and day 3. The patient was stabilized at the intensive care unit and then admitted to the internal ward on day 5. His urine output started from this time on, and AKIRisk levels could be measured. This was followed by a phase of polyuria and almost complete recovery of transplant organ function to pre-accident levels. The initially high AKIRisk of 4.7 on day 1 declined rapidly to 0.15 much earlier than did sCr, clearly indicating the restoration of transplant organ function.

### Cardiac failure

Patient #4: A 69-year-old woman with a history of diabetic nephropathy underwent cardiac bypass surgery during which she had to be resuscitated, but was eventually stabilized. Her kidney function deteriorated most likely due to infection and cardiac insufficiency, which was manifested by her decreasing urine output and rising sCr on day 3. This was paralleled by a rise in the AKIRisk score to 8.1, which dropped on day 7 after fluid substitution. SCr also fell with some delay, urine output increased, and AKIRisk reduced 2 days earlier to 0.5. Interestingly, the patient’s AKIRisk levels lagged behind the sCr increase.

Patient #5: A 77-year-old man suffered from pulmonary hypertension and failure of the right ventricle with pre-admission CKD. He also had hyponatremia and severe dyspnea. His symptoms worsened and his kidney function deteriorated, showing an elevated AKIRisk score of 5.8 on day 2. Urine flow could be reinstituted by hydration. The patient’s blood pressure rose along with renal perfusion, resulting in a marked reduction of his AKIRisk score to below 0.3. However, sCr gradually increased and the AKIRisk score rose to 5.86 on day 5, culminating in anuria, a fatal cardiac condition, and death on day 6.

Patient #6: A 70-year-old woman with morbid obesity was admitted to the hospital because of dyspnea and symptoms of cardiovascular disease. She had undergone cardiac bypass surgery 5 years earlier. After a coronary catheter intervention, 70 ml of contrast medium was administered. The patient developed atrial fibrillation and severe dyspnea the next day. This condition was associated with a rise in sCr to 6.2 mg/dl on day 1. After RRT the patient’s condition improved and her sinus rhythm was reinstituted. Her kidney function improved, as reflected by a fall in sCr as well as the AKIRisk score.

Patient #7: A 73-year-old man was admitted to the hospital for coronary catheterization prior to surgery because of severe aortic valve regurgitation and mitral valve regurgitation. He received a contrast medium on day 1 and day 2 for performing a computed tomography. For his pre-existing CKD the patient received moderate quantities of fluid pre- and post-intervention. Monitoring of urine output and sCr revealed an increase in sCr four days after initial contrast application, from 1.25 to 1.56. His AKIRisk score increased the day after the first contrast application to 1.7, and showed peak levels at 12 h after the second contrast application to 3.12, which then declined 2 days later to 1.64; the patient’s sCr fell with a 24-h delay compared to his AKIRisk score. The measurements were discontinued because the patient was discharged from the hospital.

### Severe renal failure

Patient #8: A 56-year-old woman was admitted to the hospital because of acute hepatitis due to hepatitis B virus infection. Her urine output was observed for 8 h, followed by a phase of anuria. Her AKIRisk score at the time of admission was notably high at 73; 16 h later it dropped to 62. The next day she fell into a hepatic coma and HD had to be instituted on day 3. Due to her deteriorating liver function and clinical condition, liver transplantation was performed on day 8. The patient recovered subsequently, but was unable to regain her kidney function and needed RRT until her discharge on day 44.

Patient #9: A 78-year-old patient presented with deteriorating renal function and elevated levels of C-reactive protein, which did not change even after antibiotic treatment. No therapeutic response was achieved despite adaptation of the antibiotic regimen for more than 2 months. A kidney biopsy showed granulocyte infiltration in the interstitial tissue, signs of tubular lesions, and the presence of granulocytes in the small capillaries of the glomeruli. Finally RRT had to be instituted after 2 months of hospital care, and could not be discontinued. This type of kidney disease was the sole pathological condition accompanied by a high AKIRisk score (peak 18), throughout the observation period.

### Chemotherapy-induced kidney injury

The following three case reports concern patients undergoing chemotherapy based on cisplatin. The DCF regimen, consisting of docetaxel, cisplatin and 5-fluorouracil, was administered in the following doses: docetaxel 75 mg/m^2^ day1, cisplatin 75 mg/m^2^ day1 and 5-fluorouracil 750 mg/m^2^/24 h by continuous infusion on days 1–5. About 3000 ml of fluid substitution was given along with each cisplatin administration.

Patient #10: A 41-year-old patient, initially diagnosed with gastric cancer, showed a significant rise in AKIRisk levels to 4 after the first administration of cisplatin during the first cycle on day 2 of the first cycle. This fell to below 0.5 during further treatment. Serum creatinine levels were slightly increased at the end of each cycle.

Patient #11: A 54-year-old man with esophageal cancer tolerated the regimen well. His AKIRisk score rose to higher ranges after the administration of cisplatin during each cycle, with peak levels of 2.6 on day 2 during the second cycle. A marginal increase in sCr was noted, but sCr remained in the normal range. His treatment was changed after three cycles because of cisplatin-induced neuropathy.

Patient #12: A 57-year-old man with cancer of the hypopharynx was admitted to the hospital for chemotherapy. During the first and second cycles of cisplatin his AKIRisk score increased to 2.7 on day 3 of cycle 1. Concurrently sCr was slightly elevated after the first 2 cycles, but remained within normal range.

## Discussion

The present report describes the dynamics and applicability of a novel and rapid urine-based diagnostic method for evaluating the risk of imminent kidney failure, compared to serum creatinine levels. Acute kidney injury as such is known to be a poor prognostic factor in terms of mortality [24]. We concentrated on four settings: IRI in the context of kidney transplantation, cardiac failure, severe AKI leading to end-stage renal disease, and toxin-induced kidney injury. The cases described here indicate that, in patients with ischemic reperfusion and toxic injury, [IGFBP7] · [TIMP-2] (= AKIRisk) rise rapidly and decline fast, even before polyuria becomes evident. When the AKIRisk score remains below 8, the concomitant rise in sCr and the stage of AKI appear to be reversible. Conversely, patients with values above 18 and prolonged high-levels of [IGFBP7] · [TIMP-2] excretion were unable to recover and needed renal replacement therapy permanently, as noted in patients #8 and #9. However, one 94-year-old woman with prerenal acute kidney injury and a high AKIRisk score of 17.9 recovered fully under antibiotic and hydration therapy (as shown in Additional file 3: Figure A, patient C). Another patient, also described in the supplement, had a peak level of 27 shortly after surgery, which fell to 2 over the next 32 h.

Of patients with AKI and CKD, patient #4 had a prolonged high AKIRisk score with falling sCr levels. These unexpected dynamics might be explained by the patient’s stage of CKD and her previous history of intermittent kidney replacement therapy at the intensive care unit 1 month earlier.

The course of disease seems to differ from patient to patient. As shown by other authors as well, repair mechanisms might contribute to the patients’ levels of secretion [16]. Kidney injury might be brief and quite simply caused by a surgical intervention [15], as observed in patients B and D (Additional file 3: Figure A). This may result in an increase in sCr 24 to 30 h later, while peak AKIRisk levels are observed as early as 2 h after the surgical intervention. Moreover, in cadaver kidney recipients who have experienced IRI, a rise in AKIRisk levels is seen shortly after implantation of the organ, when urine flow starts. However, these patients experience a rapid decline of values within just 48 h, which is not paralleled by polyuria. The phase of polyuria and subsequent recovery of kidney function occurred a little later than the decline in AKIRisk levels. In contrast, this was not the case when a living donor kidney was implanted, as shown in patient A (Additional file 3: Figure A). Immediately after organ implantation and urine flow, the patient’s AKIRisk score fell to below 0.06.

With regard to toxic kidney injury due to chemotherapy, the increase in AKIRisk levels was followed by a marginal increase in serum creatinine. This was noted in patient #7, whose high AKIRisk score resulted in a slight increase in serum creatinine 72 h later. The toxic effects either occur in a small population of nephrons or are of such a small magnitude that they do not result in clinically measurable pathologies. However, earlier studies have clearly shown that marginal changes in serum creatinine might be related to toxic injury, which is relevant with regard to the patient’s subsequent life span [25]. Nephrologists have claimed that renal disease might be accompanied by minimal or no changes in serum creatinine because of renal reserves and various degrees of tubular creatinine excretion [11].

The brisk kinetics of [IGFBP7] · [TIMP-2] after tubular cell injury during surgery or ischemic reperfusion was clearly seen in the majority of our patients. However, this might be different in CKD patients, especially in those with diabetic nephropathy (patient #4). Injury under these conditions might induce a more prolonged cascade of cell cycle arrest, causing signaling, which induces further fibrosis and vascular rarefication [17, 26].

The very brisk and rapid dynamics of these parameters is associated with limitations in terms of method. Timely detection of tubular cell injury might require frequent testing, especially at the intensive care unit. Further studies are needed to evaluate the applicability of the test in the very complex and varying patterns of acute kidney injury.

One of the limitations of the study and the test method is that it is still unclear why patients responding with high NephroCheck levels after chemotherapy do not show an immediate rise in sCr. However, our own observations regarding tubular-cell-derived urinary proteins show that the presence of subclinical tubular cell injury does not necessarily result in elevated sCr levels. One patient (#9) had prolonged high levels and eventually went into RRT. The secretion of [IGFBP7] · [TIMP-2] in this patient might have been connected to his renal disease.

As the present investigation was not performed for the purpose of test validation, no conclusions can be drawn in regard of risk stratification. However, the time point of NephroCheck testing is obviously crucial. Further research should be focused on the various types of disease and the corresponding AKIRisk scores.

## Conclusion

Nephrocheck was tested in 69 patients, in different settings of acute kidney injury. The dynamics of this test are compared to serum creatinine in the present report. A brisk decline in AKIRisk levels was indicative of the restoration of kidney function to a previous stage. However, the patients’ AKIRisk dynamics varied. One limitation of the study is the wide spectrum of disease entities and comorbidities in the individual patients. Future studies should be focused on specific disease entities.

## Additional files

Additional file 1:
**Table A: Demographics of the four additional patients.** (XLSX 10 kb) Additional file 2:
**Description of the four additional patients as well as table and figure legends of table A, table B, figure A, figure B.** (DOCX 18 kb) Additional file 3:
**Figure A: Time course of [IGFBP7] · [TIMP-2] of the four additional patients.** (TIF 949 kb) Additional file 4:
**Table B: Demographic data of the entire patient cohort (** ***n*** **= 69).** (XLSX 9 kb) Additional file 5:
**Figure B: TIMP-2 immunofluorescence staining of tubular cells in the urine sediment of patient #2.** (TIF 6713 kb)

## Abbreviations

IGFBP7: Insulin-like growth factor binding protein 7
TIMP2: Tissue inhibitor of metalloproteinase 2
AKI: Acute kidney injury
ESRD: End-stage renal disease
sCr: Serum creatinine
CKD: Chronic kidney disease
IRI: Ischemia reperfusion injury
TX: Transplantation
HD: Hemodialysis
RRT: Renal replacement therapy
DCF: Docetaxel- cisplatin- 5-fluorouracil
KTX: Kidney transplantation
LTX: Liver transplantation
HTX: Heart transplantation
BSL: Baseline
GFR: Glomerular filtration rate
GN: Glomerulonephritis
HTN: Hypertension
CHD: Coronary heart disease
NIDDM: Non-insulin-dependent diabetes mellitus
CTEPH: Chronic thromboembolic pulmonary hypertension
Afib: Atrial fibrillation
IDDM: Insulin dependent diabetes mellitus
CPR: Cardiopulmonary resuscitation
CABG: Coronary artery bypass graft
