# Supplementary material for: NephroCheck data compared to serum creatinine in various clinical settings
Source: BMC Nephrol. 2015 Dec 9;16:206. doi: 10.1186/s12882-015-0203-5 (PMC4674950; doi:10.1186/s12882-015-0203-5)
Supplement: Additional file 2: — Description of the four additional patients as well as table and figure legends of table A, table B, figure A, figure B. (DOCX 18 kb) [file 12882_2015_203_MOESM2_ESM.docx]

**Additional file 2**

**Case reports of the four additional patients**

**A:** A 53-year-old man with end-stage renal disease received a kidney allograft from a living donor. On day 1, prior to transplantation, his AKIRisk level was 1.4. His urine production rose immediately after implantation and his AKIRisk score fell immediately to below 0.06; sCr levels decreased consistently.

**B:** A 54-year-old patient received a cadaver liver transplant due to toxic liver cirrhosis with a Child-Pugh score of C, and was admitted to the hospital before the surgical procedure. At this time his AKIRisk score was below 0.05, but a brisk rise to 27 was observed 2 hours after surgery. This was followed by a rise in sCr 16 hours later to 1.75. The patient’s AKIRisk score decreased during observation at the intensive care unit and declined to 0.03 forty hours after surgery, while serum creatinine decreased gradually.

**C**: An 89-year-old woman was admitted to the nephrology ward in oliguric condition due to sepsis. A very high AKIRisk score (17.9) indicated that grave renal damage might have occurred. Immediate antibiotic treatment with amoxicillin/clavulanic acid and fluid substitution increased the patient’s urine output. This was associated with an intermittent rise in sCr and a rapid decline in the AKIRisk score to below 0.2. The fast drop in AKIRisk levels suggested recovery from AKI. In fact, the patient recovered fully and could be discharged from the hospital.

**D**: A 47-year-old patient received a cadaver liver transplant due to biliary cirrhosis. An AKIRisk score of 1.15 was noted shortly after transplant surgery (80 min), which dropped rapidly to below 0.2 under intensive care treatment with fluid substitution and hemodynamic stabilization. Serum creatinine levels increased over the next few days to a peak level of 3.57, but returned to pre-surgical levels at 3 weeks after transplantation.

**Figure A**: Time course of [IGFBP7]·[TIMP-2], labeled as AKIRisk on the left Y-axis (gray rectangles) and serum creatinine on the right Y-axis (black circles) in 4 patients #A-#D; sCr, serum creatinine in mg/dl; KTX, kidney transplantation; LTX, liver transplantation.

**Figure B**: TIMP-2 immunofluorescence staining of tubular cells in the urine sediment of patient #2. Cytocentrifuge preparations of urinary sediment were obtained after cell suspension in tissue culture media (RPMI 1640 supplemented with 10% newborn calf serum) using a Shandon cytospin centrifuge. Air-dried slides were fixed in acetone for five minutes. After hydration of the slide with PBS, the primary antibody (TIMP-2 (3A4): sc-21735, Santa Cruz Biotechnology, Inc.) was applied and incubated overnight at 4°C. Following two washes in PBS, the secondary antibody (donkey anti-mouse, Alexa Fluor 488, ThermoFisher Scientific) was applied and incubated for one hour at room temperature. After two washes in PBS the slide was mounted in a Vectashield mounting medium containing propidium iodide for the purpose of nuclear staining.

**Table A**: Demographics of the four patients: age, sex, baseline GFR, stage of AKI, AKIRisk peak levels during the observation period, comorbidities, and relevant surgeries before or during the observation period. Outcome data such as follow-up creatinine levels are provided, with the months of follow-up mentioned in brackets. BSL sCr, baseline serum creatinine in mg/dl; GFR, glomerular filtration rate in ml/min; AKI, acute kidney injury; sCr, serum creatinine; IRI, ischemia reperfusion injury; GN, glomerulonephritis; CKD, chronic kidney disease; HTN, hypertension; KTX, kidney transplantation; CHD, coronary heart disease; NIDDM, non-insulin dependent diabetes mellitus; CTEPH, chronic thromboembolic pulmonary hypertension; afib, atrial fibrillation; IDDM, insulin dependent diabetes mellitus; HTX, heart transplantation; CPR, cardiopulmonary resuscitation; CABG, coronary artery bypass graft surgery; LTX, liver transplantation.

**Table B**: Demographic data of the entire patient cohort (n=69). The nephrology cohort (n=52) and the oncology cohort (n=17) are shown separately. Patients were divided into three groups, based on their AKIRisk scores (<0.3; 0.3-2; >2). For each group, the number of patients in different stages of AKI is indicated. In the nephrology group, NC (non-classified) indicates kidney recipients who had been on dialysis before transplantation.
