# Supplementary figures and images for: NephroCheck data compared to serum creatinine in various clinical settings
Source: BMC Nephrol. 2015 Dec 9;16:206. doi: 10.1186/s12882-015-0203-5 (PMC4674950; doi:10.1186/s12882-015-0203-5)

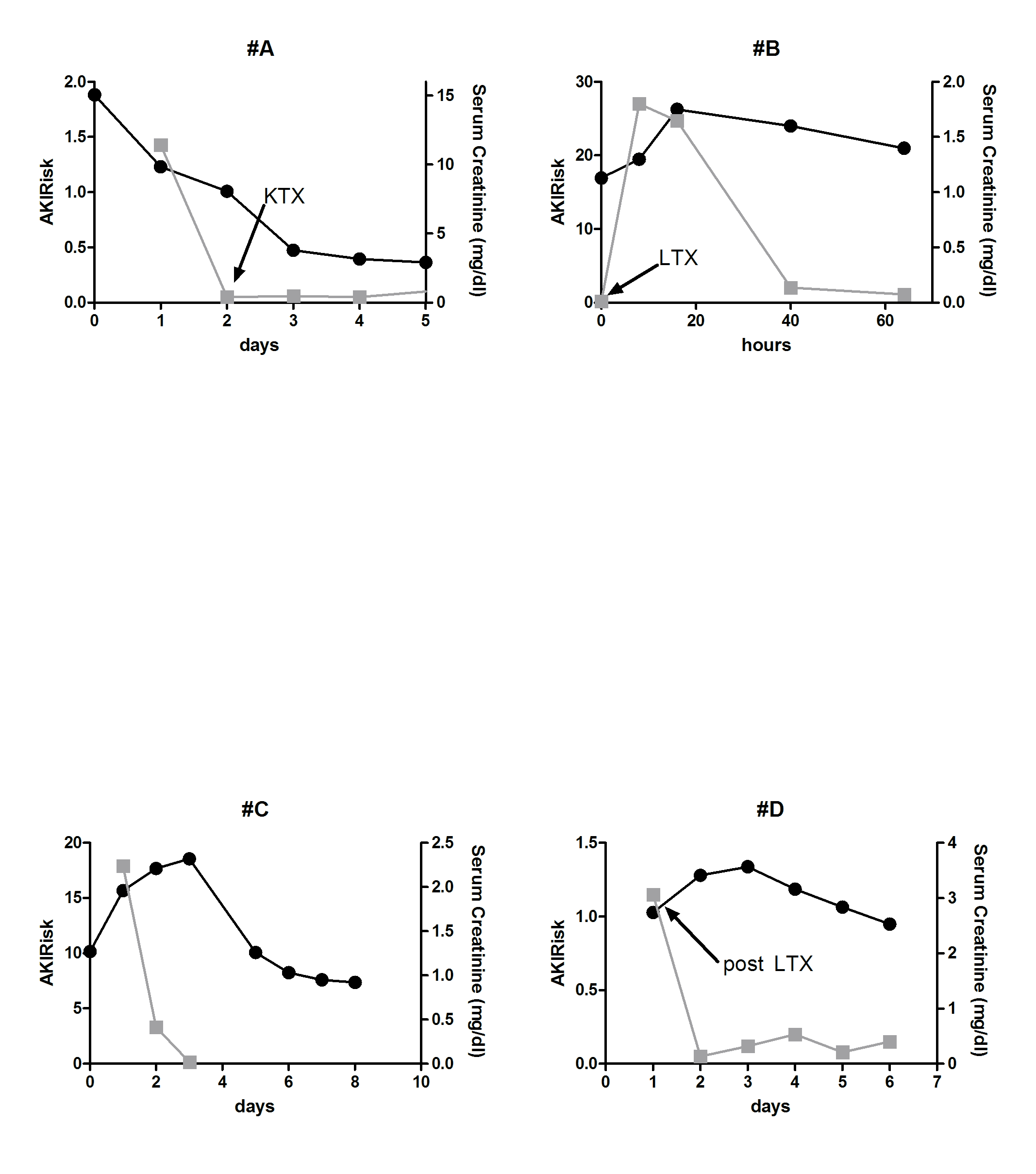

Supplement: Additional file 3: — Figure A: Time course of [IGFBP7] · [TIMP-2] of the four additional patients. (TIF 949 kb) [file 12882_2015_203_MOESM3_ESM.tif]

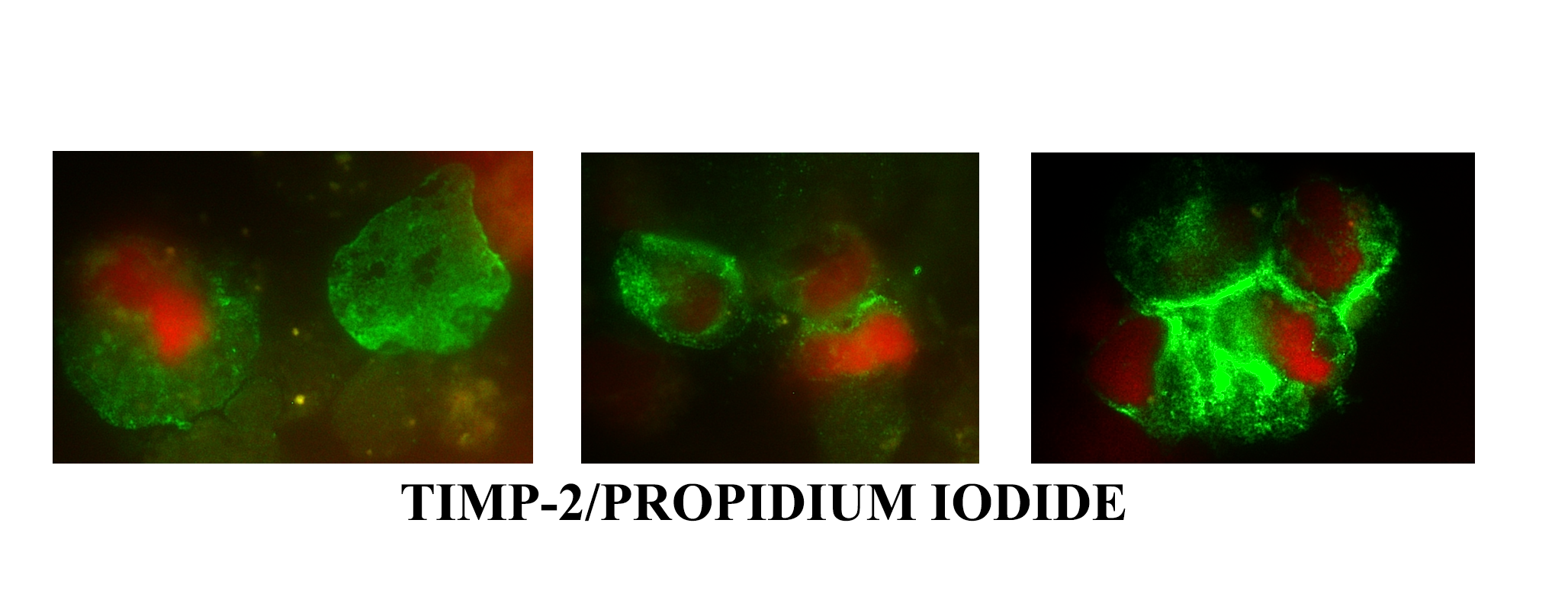

Supplement: Additional file 5: — Figure B: TIMP-2 immunofluorescence staining of tubular cells in the urine sediment of patient #2. (TIF 6713 kb) [file 12882_2015_203_MOESM5_ESM.tif]
